# Are heat waves susceptible to mitigate the expansion of a species progressing with global warming?

**DOI:** 10.1002/ece3.690

**Published:** 2013-07-30

**Authors:** Christelle Robinet, Jérôme Rousselet, Patrick Pineau, Florie Miard, Alain Roques

**Affiliations:** INRA, UR633, Zoologie ForestièreF-45075 Orléans, France

**Keywords:** Climate change, climatic anomaly, egg, forest pest, heat wave, insect, pine processionary moth, solar radiation, temperature, *Thaumetopoea pityocampa*

## Abstract

A number of organisms, especially insects, are extending their range in response of the increasing trend of warmer temperatures. However, the effects of more frequent climatic anomalies on these species are not clearly known. The pine processionary moth, *Thaumetopoea pityocampa*, is a forest pest that is currently extending its geographical distribution in Europe in response to climate warming. However, its population density largely decreased in its northern expansion range (near Paris, France) the year following the 2003 heat wave. In this study, we tested whether the 2003 heat wave could have killed a large part of egg masses. First, the local heat wave intensity was determined. Then, an outdoor experiment was conducted to measure the deviation between the temperatures recorded by weather stations and those observed within sun-exposed egg masses. A second experiment was conducted under laboratory conditions to simulate heat wave conditions (with night/day temperatures of 20/32°C and 20/40°C compared to the control treatment 13/20°C) and measure the potential effects of this heat wave on egg masses. No effects were noticed on egg development. Then, larvae hatched from these egg masses were reared under mild conditions until the third instar and no delayed effects on the development of larvae were found. Instead of eggs, the 2003 heat wave had probably affected directly or indirectly the young larvae that were already hatched when it occurred. Our results suggest that the effects of extreme climatic anomalies occurring over narrow time windows are difficult to determine because they strongly depend on the life stage of the species exposed to these anomalies. However, these effects could potentially reduce or enhance the average warming effects. As extreme weather conditions are predicted to become more frequent in the future, it is necessary to disentangle the effects of the warming trend from the effects of climatic anomalies when predicting the response of a species to climate change.

## Introduction

Climate change is currently affecting many insect species and a large variety of responses have already been detected (e.g., Bale et al. 2002; Hill et al. 2002; Parmesan 2006; Netherer and Schopf 2010; Robinet and Roques 2010). Most of the studies conducted until now have focused on the effects of the warming trend. However, climatic anomalies are predicted to be more frequent in the future (Meehl and Tebaldi 2004; Beniston et al. 2007; Meehl et al. 2007; Coumou and Rahmstorf 2012). Heat waves have become increasingly frequent since the 1950s at global as well as continental scales, especially in North America, Europe, and Australia, and their frequency and magnitude are predicted to continue increasing along the 21st century (Easterling et al. 2000; Meehl et al. 2007; Seneviratne et al. 2012). The last 10 years were the hottest recorded in Europe since the 1500s, and the 2003 heat wave was exceptional over western and central Europe (Rebetez et al. 2006; Barriopedro et al. 2011). A few studies intended to assess the effects of the 2003 heat wave on plant growth and productivity (e.g., Ciais et al. 2005; Rennenberg et al. 2006), insect flight (Battisti et al. 2006; Hochkirch and Damerau 2009), and forest insect dynamics (for a review see Rouault et al. 2006). However, it is still unclear whether this weather anomaly may enhance or mitigate the effects of the current warming trend on the expansion process that is observed in several plant pests.

**Figure 1:**
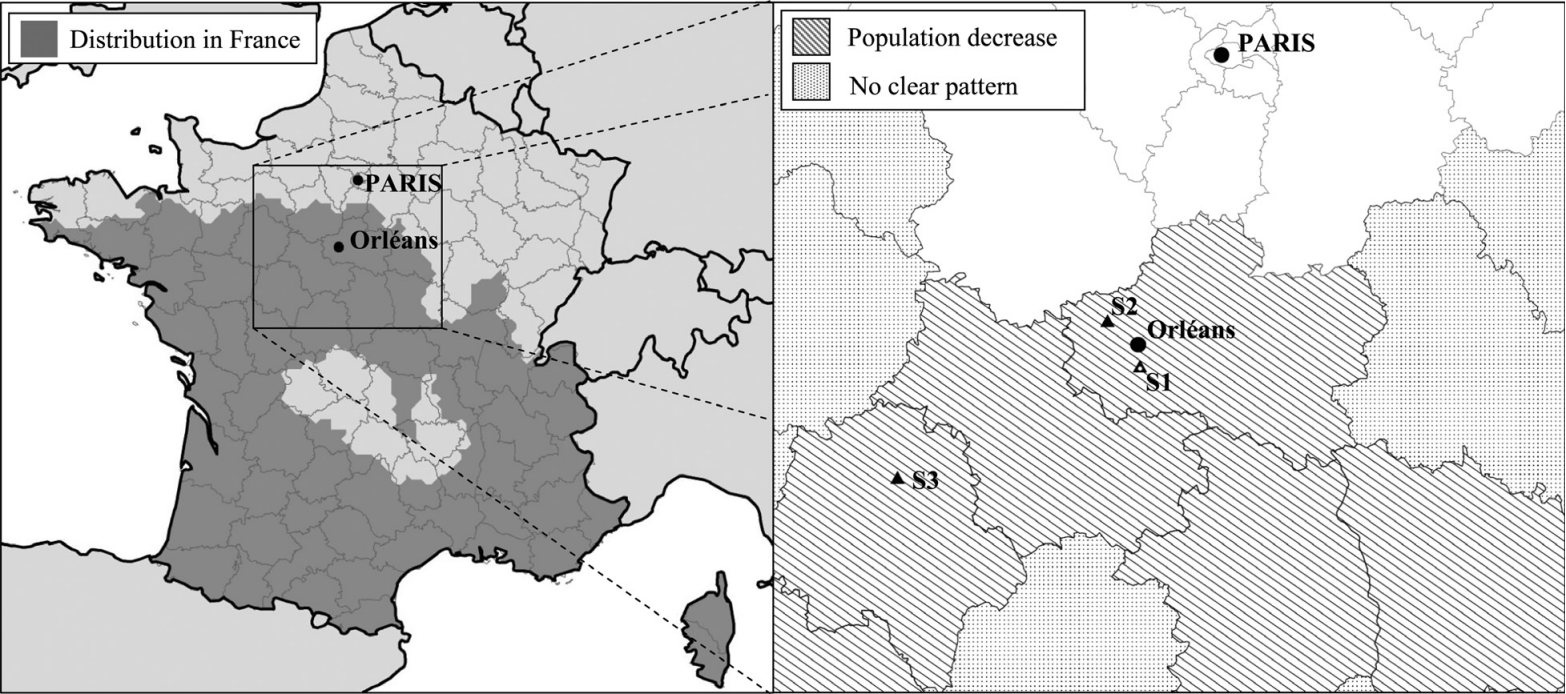
The study area is the northern distribution of the pine processionary moth in France (region of Orléans, located in southern Paris Basin). Dark gray indicates the distribution during the winter 2005–2006 (derived from Robinet et al. 2010). Black triangles indicate the location of weather stations: S1 is the station located on the INRA campus and the white dot indicates where the outdoor experiment was done, S2 is the station near Orléans, and S3 is the station at Parçay–Meslay. The background on the right figure represents the change in the population density between the winters 2002–2003 and 2003–2004 in the French administrative entities (derived from Bouhot-Delduc 2005). The population level uniformly decreased over hatched entities, whereas the change was unclear over dotted entities.

The range expansion of the pine processionary moth, *Thaumetopoea pityocampa*, is acknowledged to be directly associated with the recent climate warming up (Rosenzweig et al. 2007). In this species whose larvae are developing during winter, the warming-up trend in winter temperatures observed in Europe results in conditions more and more favorable for the feeding activity and survival of larvae (Battisti et al. 2005; Robinet et al. 2007; Hoch et al. 2009). Consequently, the pine processionary moth is extending its geographical distribution northward and at higher elevations (Fig. 1; Battisti et al. 2005). In France, the moth has thus continuously progressed northward in the southern basin of Paris since the mid-1990s, at an average speed of ca. 5 km per year (Battisti et al. 2005). However, during autumn 2003, following the summer heat wave with maximal temperatures reaching around 40°C, a significant collapse was noticed in the number of new larval colonies at the northern edge of this expansion range, in the region of Orléans, near Paris (Bouhot-Delduc 2005; Fig. 1). This case study thus offers a very good example to understand whether a climatic anomaly, the 2003 heat wave, may have affected the moth's current expansion.

Several hypotheses can be made to explain the decrease in density of the pine processionary moth in its northern range following the 2003 heat wave: (1) adult emergence may have been disturbed by unusually high temperatures, and thus perturbing sex encountering and decreasing mating success; (2) high temperatures may have killed a large part of egg masses; (3) high temperatures may have killed a large part of young larvae; and (4) heat and drought may have affected the quality of the needles resulting in a decrease in the survival rate of the larvae. Among all these hypotheses, (2) and (3) were usually considered by experts in forest health (DSF Nord-Ouest 2004; Bouhot-Delduc 2005; Piou et al. 2006) probably because temperatures higher than 32°C are supposed to have strong negative effects on incubating eggs and larvae (Huchon and Démolin 1970). A population collapse due to a strong effect on egg development would be an explanation consistent with sparse field observations reporting an apparent absence of feeding damages caused by neonates in August.

However, until now, no study has been carried out to verify these hypotheses. In this study, we intended to test hypothesis (2) because some basic evidence supported an effect on egg masses. A large proportion of eggs are usually laid in midsummer at the northern edge of the pine processionary moth distribution, and therefore, this life stage was probably the most exposed to the 2003 heat wave. Moreover, egg masses are usually laid near the apex of branches which are sun exposed. As insects that are sunlight exposed may experience higher temperatures (Pincebourde et al. 2007), egg masses probably experienced temperatures even higher than those recorded by weather stations during the 2003 heat wave.

Our study thus aimed at exposing processionary egg masses to artificial heat wave conditions mimicking those encountered during summer 2003 in the northern expansion range (Orléans region), in order to compare egg survival and larval development until the third instar with regard to those observed under control conditions represented by the average daily temperatures recorded in July–August over the period 1973–2002. Larvae are not urticating until the third instar, which corresponds to the stage at which most larvae enter winter in the expansion range.

## Material and Methods

### Characterization of the temperature conditions during the 2003 heat wave in North-Central France

Daily maximum and minimum temperatures recorded by a weather station near Orléans (47.98°N 1.75°E, ca. 20 km from INRA campus; Fig. 1, Station S2) were retrieved from the European Climate Assessment and Dataset (Tank et al. 2002). The magnitude of the 2003 heat wave (in terms of high temperatures but also in terms of duration) was assessed with regard to the temperature observed during the preceding 30 years (1973–2002) at the same place. The number of consecutive days where the maximum temperature was above 32°C was calculated. As heat waves can be defined by unusually high maximum temperatures but also by unusually high minimum temperatures (Bessemoulin et al. 2004), the number of consecutive days during which the minimum temperature was above 18°C was also measured. The calculation is based on this threshold temperature, 18°C, because it is the highest minimum temperature ever recorded in Orléans between 1973 and 2002 in early August.

### Measurement of the temperature inside egg masses

Fourteen egg masses of pine processionary moth were collected on different trees (1 mass per tree) in the Orléans region during the usual peak of adult emergence in July 2011. All of them were installed on the same sun-exposed pine (*Pinus sylvestris*), ca. 3 m tall, in the INRA campus at Orléans (47.82°N 1.91°E; Fig. 1). Female usually lay egg masses around a couple of needles, but these needles were removed for the experiment, and instead a thermocouple probe was inserted inside the egg mass. Each thermocouple was connected to a CR10X data logger (Campbell Scientific Inc., Logan, UT), which was placed on the ground near the base of the pine trunk. This data logger recorded temperature within each egg mass every 5 min. Each day, the probes were checked to remain still inside the middle of the egg masses. The hatching of the egg masses was checked at the same time. When egg masses hatched, they were simply removed, the temperature record was stopped, and in the analysis we only kept temperature data until the previous check (to be sure to really consider the temperature within unhatched egg masses).

In parallel, hourly air temperature (°C) and solar radiation (Joule/cm²) were obtained from a weather station located at INRA campus (data accessed from the INRA Climatik platform, https://internet.inra.fr/climatik/; Fig. 1, Station S1), at around 400 m from the experiment site. To fit the temporal resolution used in the experiment, the temperature and solar radiation recorded each hour (e.g., 01:00 pm) were supposed to be constant over the following hour, and thus data at 5-min time steps were obtained (e.g., 01:00–01:55 pm under the same weather conditions). A linear regression was applied in order to estimate the temperature inside sun-exposed egg masses with regard to the temperature and solar radiation recorded by the weather station.

As this station was not present on the campus in 2003 and the closest one (Station S2, Fig. 1) did not record solar radiation at that time, hourly temperature and solar radiation recorded in August 2003 at Parçay–Meslay (47.45°N 0.73°E, ca. 90 km west from Orléans; Fig. 1, Station S3) were used (data accessed from INRA Climatik platform). This weather station is also located in a region where the population level decreased (Fig. 1; Bouhot-Delduc 2005). A regression was applied to this data set to estimate the temperature that the egg masses exposed to the sun could have faced in 2003.

Additionally, assuming that solar radiation was similar at both locations, we used the daily maximum solar radiation in Parçay–Meslay (S3) (calculated from the hourly Climatik data set) and the daily maximum temperature near Orléans (S2) (obtained from the European Climate Assessment and Dataset; Tank et al. 2002) to estimate the maximum temperature inside sun-exposed egg masses in Orléans during the heat wave in 2003.

### Effect of an artificial heat wave on egg masses

**Figure 2:**
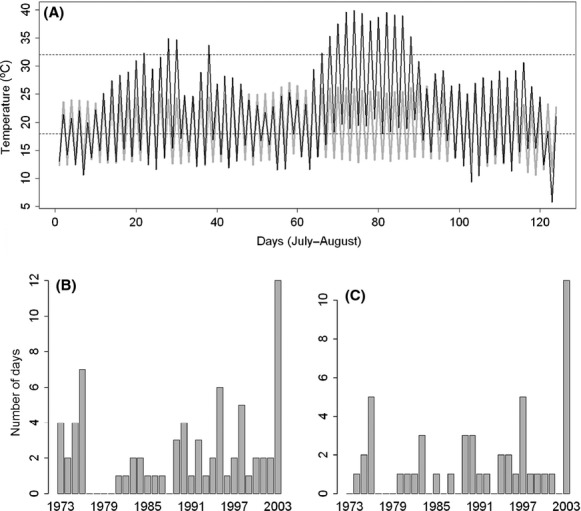
(A) Anomaly of temperature in Orléans in 2003. The gray line represents the mean temperature over 1973–2002 and the black line the mean temperature in 2003, between 1st July and 31th August. The upper dashed line indicates a temperature of 32°C, and the lower one, a temperature of 18°C. (B) Number of consecutive days where the daily maximum temperature was above 32°C. (C) Number of consecutive days where the daily minimum temperature was above 18°C.

A total of 103 egg masses were collected in July 2010 in Orléans. These egg masses were sequentially placed in climatic chambers to simulate daily minimum temperature and in incubators to simulate daily maximum temperature according to the following design (Table 1). Each day, the egg masses were removed from climatic chambers at 11:30 am, placed in incubators which automatically started to heat at that time, and then removed at 5:30 pm to be placed again in climatic chambers, and so on. This timing was chosen to mimic the daily temperature cycle outdoors. Hereafter, “night” will refer to the period between 5:30 pm and 11:30 am, and “day” to the period between 11:30 am and 5:30 pm. Under the control conditions (called T24), egg masses were placed at 13°C in climatic chambers during the night and at 24°C in incubators during the day. These temperatures represent the historical average of daily minimum and maximum temperatures from July to August over the period 1973–2002 (Fig. 2A). In the two heat wave conditions (called T32 and T40), egg masses were all placed at 20°C in climatic chambers during the night, and at 32–40°C in incubators during the day.

**Table 1:** Description of heat wave treatments: the daily temperature cycle was based on the following N/D temperatures for each treatment (N, “night” from 5:30 pm to 11:30 am; D, “day” from 11:30 am to 5:30 pm)

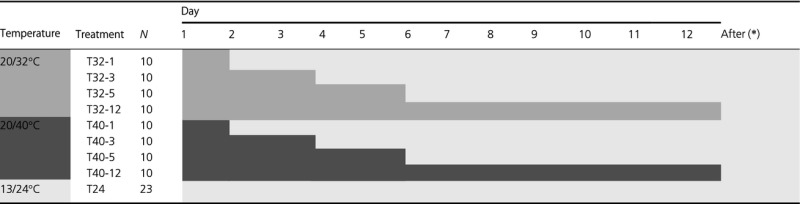

The treatment simulating a strong heat wave (20/40°C) is represented in dark gray, the treatment simulating a moderate heat wave (20/32°C) is represented in moderate gray, and the control treatment (13/20°C) is represented in light gray. After heat wave treatments, egg masses were then placed in the control treatment until egg hatching (*). N is the number of egg masses exposed to the treatment.

Several durations for the heat wave conditions were also considered: 1 day (T32-1 and T40-1), 3 days (T32-3 and T40-3), 5 days (T32-5 and T40-5), or 12 days (T32-12 and T40-12). All the egg masses were placed under these conditions at the same time, but as soon as the given duration was reached, the egg masses were placed under the control conditions (T24). Egg masses were thus transferred daily from climatic chambers to incubators until their hatching, and then larvae were reared in the laboratory under ambient temperature (minimum: 15.6°C, mean: 20.7°C, and maximum: 23.8°C) until they reach the third instar. This instar was considered because the first instars are the most sensitive to abiotic pressures (Santos et al. 2011). Moreover, the larvae then become urticating and they are more difficult to rear in laboratory. Immediately after hatching, the larvae were fed every day with 10 cm long twigs of *Pinus sylvestris* freshly cut on INRA campus. At the end of this experiment, the following variables were measured: number of eggs per egg mass, number of hatched eggs, number of parasitized eggs, number of empty eggs, number of “yellow” eggs (undeveloped eggs with dried-up yolk), and number of eggs with a dead larva inside. These egg classes are derived from classes usually found for the processionary moths in Europe (see for instance Tsankov et al. 1996). All these numbers were converted into a percentage of eggs per egg mass. Furthermore, to detect a potential delayed effect of high temperatures on the larval development, the number of larvae reaching the third instar per egg mass was counted. We compared the results from the different heat wave treatments based on these data. As these percentages were not normally distributed, we used the nonparametric Kruskal–Wallis test.

## Results

### Characterization of the temperature conditions during the 2003 heat wave in North-Central France

The temperature was considerably higher than usual from August 2nd to 13th (Fig. 2A). The maximum temperature was 10–14°C higher than usual maximum temperatures, and minimum temperature was 4–7°C higher than usual minimum temperatures for 11 consecutive days. The maximum temperature was between 32°C (lethal temperature of eggs and young larvae reported by Huchon and Démolin 1970) and 40°C during 12 consecutive days (Fig. 2A, B). Moreover, the minimum temperature was above 18°C during 11 consecutive days (Fig. 2A, C). This duration of heat wave was exceptional compared to the previous years (Fig. 2 B, C). The maximum temperature rarely reached this 32°C threshold over several consecutive days and, in any case, it has never lasted more than 7 consecutive days since 1973 (Fig. 2B). Similarly, the minimum temperature rarely reached the 18°C threshold over several days, and in any case, it has never lasted more than 5 days (Fig. 2C). The number of consecutive days with temperature above these two thresholds (daily maximum temperature above 32°C and daily minimum temperature above 18°C) was the highest in 2003, reaching 10 days.

### Measurement of the temperature within egg masses

A large part (88%) of the variation in the temperature within sun-exposed egg masses was explained by the following regression (*F*_2,10,138_ = 36,120, *P* < 0.001):


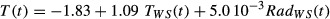


where *T*(*t*) is the temperature within sun-exposed egg masses at time *t*, 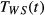 and 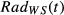 are respectively, the temperature and solar radiation recorded at time *t* by the weather station on the INRA campus. All *P*-values were highly significant (*P* < 0.001).

**Figure 3:**
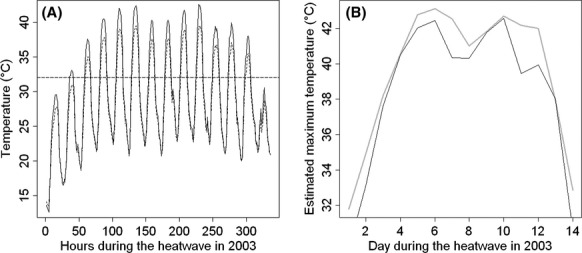
(A) Hourly temperature recorded by the weather station in Parçay–Meslay during the heat wave in 2003 (dashed black curve) and estimated temperature inside sun-exposed egg masses (continuous black curve). The horizontal dashed line indicates the 32°C threshold. (B) Estimated daily maximum temperature inside sun-exposed egg masses in Parçay–Meslay (black curve) and in Orléans (thick gray curve) from 1st to 14th August 2003. This second temperature time series was reconstructed using daily maximum solar radiation recorded in Parçay–Meslay and daily maximum temperature recorded in Orléans.

When this regression was applied to the temperature and solar radiation recorded in 2003 by the weather station in Parçay–Meslay (S3), the estimated temperature within sun-exposed egg masses was above the 32°C threshold during 12 days and reached 42.6°C on 10th August (Fig. 3). The daily maximum temperature inside sun-exposed egg masses was on average 2.8°C higher than the temperature given by the weather station during this heat wave (1st–14th August 2003). When estimating the daily maximum temperature in sun-exposed egg masses in Orléans, temperatures were slightly higher than in Parçay–Meslay, above 32°C during 13 consecutive days, above 42°C during 6 days (two series of three consecutive days), and reached a maximum of 43.1°C on 6th August 2003 (Fig. 3B).

### Effect of an artificial heat wave on egg masses

**Figure 4:**
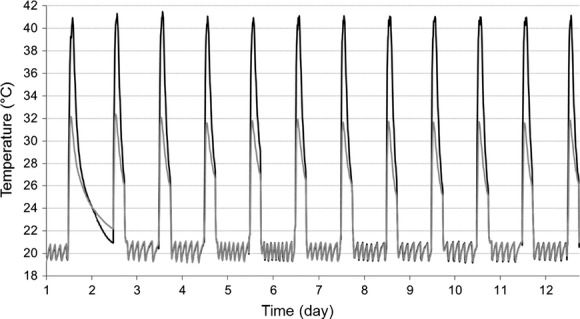
Temperature actually recorded in incubators and climatic chambers during 12 days (the maximum duration of heat wave treatments) for T32 (gray curve) and T40 (black curve).

The temperature level in incubators and climatic chambers for the T40 treatment mimicked relatively well the heat wave which occurred in Orléans in 2003 (Fig. 4). Daily maximum temperature was effectively around 41°C for T40 and around 32°C for T32, and daily minimum temperature was around 20°C.

Only 9 of 103 egg masses showed no egg hatching at all, and they were uniformly distributed over the treatments (Table 2). These unhatched egg masses mostly contained yellow eggs, the others being either parasitized or empty. Thus, they were removed from the analysis for which we only kept egg masses in which at least one egg hatched. The percentage of hatched eggs (*χ*² = 10.69, df = 9, *P* = 0.30), parasitized eggs (*χ*² = 6.91, df = 9, *P* = 0.65), empty eggs (*χ*² = 15.00, df = 9, *P* = 0.09), yellow eggs (*χ*² = 13.38, df = 9, *P* = 0.15), and eggs with dead larva (*χ*² = 9.40, df = 9, *P* = 0.40) was not significantly different among the treatments (Table 2). The percentage of eggs resulting in alive third instar larvae did not differ significantly between treatments (*χ*² = 9.97, df = 9, *P* = 0.35).

**Table 2:** Results of the artificial heat wave experiment

Treatment	*N*	*N*_hatch_	*E*^1^	*E*_hatch_^1^^,^^2^	*E*_par_^1^^,^^2^	*E*_empty_^1^^,^^2^	*E*_yellow_^1^^,^^2^	*E*_dead_^1^^,^^2^	*L3*^1^^,^^2^
T24	23	22	230 (47)	159 (59)	31 (37)	0 (1)	35 (29)	2 (3)	150 (57)
T32-1	10	9	204 (47)	172 (45)	11 (14)	1 (1)	11 (10)	4 (5)	159 (40)
T32-3	10	8	212 (65)	137 (77)	25 (29)	0 (0)	39 (62)	5 (11)	131 (78)
T32-5	10	9	220 (50)	172 (76)	25 (38)	0 (0)	19 (26)	1 (1)	152 (62)
T32-12	10	9	214 (44)	191 (44)	14 (23)	0 (1)	6 (6)	2 (3)	156 (52)
T40-1	10	9	208 (39)	158 (62)	16 (25)	0 (1)	29 (28)	1 (1)	138 (57)
T40-3	10	9	218 (44)	146 (52)	30 (34)	0 (0)	42 (58)	2 (2)	130 (46)
T40-5	10	10	226 (51)	191 (63)	14 (17)	0 (1)	17 (13)	3 (4)	180 (55)
T40-12	10	9	229 (35)	158 (86)	14 (23)	0 (0)	49 (79)	1 (1)	145 (83)

*N* is the number of egg masses, *N*_hatch_ is the number of egg masses from which at least one egg hatched, *E* is the number of eggs per egg mass, *E*_hatch_ is the number of hatched eggs per egg mass, *E*_par_ is the number of parasitized eggs per egg mass, *E*_empty_ is the number of empty eggs per egg mass, *E*_yellow_ is the number of yellow eggs per egg mass, *E*_dead_ is the number of eggs with dead larva per egg mass, and *L3* is the number of third instar larvae per egg mass.

1 Means that the value is the mean per egg mass and standard deviation is given in brackets.

2 Means that calculations were done only on egg masses from which at least one egg hatched.

## Discussion

Regarding the complexity of insect response to climatic anomalies but also the stochasticity and unpredictability of such anomalies, and their localized occurrence in time and space, a wide range of experiments can be conducted (Jentsch et al. 2007). The approach that has been used here but also in other recent studies (e.g., Sorte et al. 2010; Gillespie et al. 2012; Landis et al. 2012; Sentis et al. 2013) is the simulation of heat wave conditions in laboratory, with different severity levels. Although this method does not allow reproducing exactly what happens outdoors, it allows exploring some mechanisms.

This study gives evidence that the eggs can survive at 40°C. Both temperature and exposure duration were tested, but it was not possible to control the age of egg masses as they were collected in the field. The age of egg masses could possibly affect their tolerance to unusually high or low temperatures (e.g., older eggs of the cactus moth, *Cactoblastis cactorum*, are more resistant to cold temperatures, McLean et al. 2006). We collected egg masses at the beginning of the adult emergence to have a lower variability in the egg age and we randomly assigned the egg masses to the treatments, but the role of this factor needs further investigation.

The maximal temperature tested in the heat wave simulation is derived from the temperature estimated inside egg masses during the 2003 heat wave. Based on air temperature and solar radiation, we were able to estimate this temperature and find that temperature inside egg masses probably exceeded the air temperature by around 5°C during the heat wave (Fig. 3A). Our results are globally in agreement with Milani (1990) who found that the temperature inside sun-exposed egg masses was higher than the air temperature and closely associated with solar radiation. The variability in the temperature difference observed in our study is, however, higher (the difference being between −11°C at dawn and +10°C in midafternoon vs. between +1.1°C and +14°C for Milani). The temperature was continuously recorded over around 1 month every 5 min (*N* = 14 egg masses) versus 3 h every second (*N* = 3 egg masses). Our study is perhaps more representative of the overall effects along the incubation period of the egg masses, whereas the study of Milani (1990) was designed to better understand the mechanism during a short period and may not be representative of the variability over the incubation period and among egg masses.

The upper temperature threshold is undoubtedly much higher than the threshold of 32°C previously reported by Huchon and Démolin (1970). Whether the value was underestimated or it corresponds to an adaption to global warming in the expanding populations is not known. These results do not mean that the heat wave had no effects on the pine processionary moth as its population level was effectively reduced the year after in a clearly delimitated area (Fig. 1). It cannot be totally excluded that the population level decreased during the winter 2003–2004 independently of this heat wave as there could be a natural outbreak cycle (Robinet 2006). However, a similar collapse appeared to have affected populations which were at different phases of their outbreak cycle in southern Paris Basin (Fig. 1) (Bouhot-Delduc 2005; Hubert Pauly, from the Forest Health Department of the French Ministry of Agriculture, personal communication). This region is located in a zone where the heat wave was the most intense in terms of number of days with maximum temperature above 40°C (Bessemoulin et al. 2004). Therefore, the heat wave had undoubtedly some effects on the populations of the pine processionary moth and other hypotheses should be considered. The first one is that adult emergence might have been disturbed by unusually high temperatures and mating success subsequently decreased. This hypothesis can be rejected because the largest capture of male moths by pheromone traps was observed in early July 2003, and then the captures decreased almost linearly until late August 2003 (J. Rousselet, unpubl. data). This trapping pattern, apparently typical for the region (see Fig. 13 in Abgrall 2001), suggests a flight period, and a subsequent mating period, concentrated during early summer, that is, before the heat wave. Therefore, it is likely that the heat wave could not really increase mating failures and decrease the overall reproduction success. Another hypothesis is that high temperatures observed during the heat wave killed a large part of the early-instar larvae. It takes about 30–45 days for the eggs to hatch (Huchon and Démolin 1970). Because the flight was observed to peak in early July, and the egg-laying period thus assumed to occur just close, a nonnegligible proportion of eggs could have developed into larvae by 1–15 August. As it is known that young larvae (first and second instar larvae, especially neonates) are very sensitive to high temperatures (Santos et al. 2011), the decrease in the population density can possibly be explained by high mortality of young larvae during the heat wave. The last hypothesis is that the heat and drought affected the quality of the needles and, hence, the survival rate of the larvae decreased, but to our knowledge, there are no data to explore this hypothesis until now. In conclusion, it is more likely that the heat wave affected directly or indirectly young larvae instead of eggs or adults. With more frequent heat waves in the future, the population abundance could be reduced. Although there is no clear evidence so far about the overall effect, higher variability in summer temperatures may perhaps mitigate the positive effect of the mean increase in winter temperature. Data collection is needed in the coming years to explore more precisely this contrasting effect of climate warming.

As a consequence of repeated heat waves, it may also be possible that the pine processionary moth will adapt its phenological cycle. Such adaptations to the specific weather conditions of each biogeographic region have been noticed for a long time (Huchon and Démolin 1970). Indeed, the adults fly in autumn in the southern range and at low elevations, avoiding high summer temperatures, whereas they fly in summer in the northern range and at high elevations, facing generally moderate summer temperatures. In addition, the moth has also the capacity to adapt its phenological cycle to new conditions in a given region such as the summer population in Portugal whose adults fly in spring (Santos et al. 2007, 2011). Therefore, possible adaptations of the phenological cycle should also be carefully checked for in the following years.

A large amount of studies have been conducted to determine the cold tolerance of insect species and the relaxation of the cold limitation in the context of climate warming (e.g., Ungerer et al. 1999; Crozier 2003; Hoch et al. 2009). Regarding heat tolerance, most of the studies have focussed on the efficiency of quarantine treatments, such as for fruit flies (e.g., Yahia and Ortega-Zaleta 2000) or codling moths (e.g., Wang et al. 2004). Unfortunately, little is known about the heat resistance of insect species in the context of more frequent heat waves. Species with a great heat tolerance could have a low potential of acclimation (Hazell et al. 2010) and have already reached their limits, and therefore heat waves could induce considerable mortality. The extent of the plasticity in these traits and possible acclimation are therefore important to determine the effects of heat waves on species range expansion.

Warming-induced range expansion is a complex mechanism and other factors than survival are involved. For instance, wing morph expression or flight activity can be associated with temperature and thus heat waves could accelerate the range expansion (Battisti et al. 2006; Hochkirch and Damerau 2009). Because the phenological development of processionary moth is delayed at higher altitude, the 2003 heat wave occurred when moths were mostly at the adult stage in the Italian Alps. Battisti et al. (2006) showed that the colonies expanded in altitude during the following winter at a ten times faster speed than previously recorded because of an increased flight activity of females during the heat wave. This mechanism was not observed in its northern range because most of the adults (flying stage) emerged well before the heat wave, and thus only a very small proportion of the adults were exposed to these temperatures. This example outlines the importance to determine precisely the life stage of the individuals exposed to heat waves in addition to the spatial heterogeneity of such extreme events. It also raises the question of the factor(s) that can explain the susceptibility of the species to heat waves beyond heat tolerance. For instance, do insect species at the adult or egg stage during summer have lower probability to suffer from heat waves than the larval stage?

This study contributes to a better understanding of the effects of a heat wave on a well-known species but also highlights the complexity of the species that answer to climate change. As extreme weather conditions are predicted to become more frequent, the challenge consists now in disentangling the effects of the warming trend from the effects of climatic anomalies when predicting the answers of species to climate change.
